# Giant piezoresistivity in a van der Waals material induced by intralayer atomic motions

**DOI:** 10.1038/s41467-023-37239-9

**Published:** 2023-03-18

**Authors:** Lingyun Tang, Zhongquan Mao, Chutian Wang, Qi Fu, Chen Wang, Yichi Zhang, Jingyi Shen, Yuefeng Yin, Bin Shen, Dayong Tan, Qian Li, Yonggang Wang, Nikhil V. Medhekar, Jie Wu, Huiqiu Yuan, Yanchun Li, Michael S. Fuhrer, Changxi Zheng

**Affiliations:** 1 School of Physics and Optoelectronics, South China University of Technology, Guangzhou, Guangdong, 510641 China; 2 Department of Materials Science and Engineering, & ARC Centre of Excellence in Future Low Energy Electronics Technologies, Monash University, Clayton, VIC Australia; 3 Key Laboratory for Quantum Materials of Zhejiang Province, School of Science, Westlake University, Hangzhou, 310024 Zhejiang Province China; 4 Center for Correlated Matter and Department of Physics, Zhejiang University, Hangzhou, China; 5 Guangzhou Institute of Geochemistry, Chinese Academy of Sciences, 510640 Guangzhou, Guangdong China; 6 Shandong Key Laboratory of Optical Communication Science and Technology, School of Physics Science and Information Technology, Liaocheng University, Liaocheng, 252000 China; 7 Center for High Pressure Science and Technology Advanced Research (HPSTAR), Beijing, 100094 China; 8 Institute of High Energy Physics, Chinese Academy of Sciences, Beijing, 100049 China; 9 ARC Centre of Excellence in Future Low-Energy Electronics Technologies, & School of Physics and Astronomy, Monash University, Melbourne, VIC 3800 Australia

**Keywords:** Electronic properties and materials, Two-dimensional materials

## Abstract

The presence of the van der Waals gap in layered materials creates a wealth of intriguing phenomena different to their counterparts in conventional materials. For example, pressurization can generate a large anisotropic lattice shrinkage along the stacking orientation and/or a significant interlayer sliding, and many of the exotic pressure-dependent properties derive from these mechanisms. Here we report a giant piezoresistivity in pressurized *β*′-In_2_Se_3_. Upon compression, a six-orders-of-magnitude drop of electrical resistivity is obtained below 1.2 GPa in *β′*-In_2_Se_3_ flakes, yielding a giant piezoresistive gauge *π*_*p*_ of −5.33 GPa^−1^. Simultaneously, the sample undergoes a semiconductor-to-semimetal transition without a structural phase transition. Surprisingly, linear dichroism study and theoretical first principles modelling show that these phenomena arise not due to shrinkage or sliding at the van der Waals gap, but rather are dominated by the layer-dependent atomic motions inside the quintuple layer, mainly from the shifting of middle Se atoms to their high-symmetric location. The atomic motions link to both the band structure modulation and the in-plane ferroelectric dipoles. Our work not only provides a prominent piezoresistive material but also points out the importance of intralayer atomic motions beyond van der Waals gap.

## Abstract (web summary)

Lattice shrinkage is a dominating factor for the strain-induced change of the electronic properties in vdW layered materials. Here, the authors discover a piezoresistivity in pressurized β′-In2Se3, which originates from the intralayer atomic motions.

## Introduction

Van der Waals (vdW) layered crystals are a class of materials featuring with weak interlayer coupling owing to the absence of strong chemical bonds in the vdW gap. Many important scientific phenomena such as superconductivity, quantum anomalous Hall effect, and interlayer exciton condensate may emerge by using gap engineering methods, e.g., interlayer twisting, artificial multilayers stacking, and atom/molecular intercalation^1–4^. Besides these methods, hydrostatic pressurization is another attractive technique for material property modifications due to its capability of driving materials to metastable states unstable at ambient pressure^5^. Meanwhile, understanding the effect of pressure induced strain on the electronic properties of vdW materials is of great importance to the development of emerging strain-based electronics such as strain gauge, electronic skin, and robotics^6^. Thus, intensive efforts have been devoted to the studies of pressure induced electronic phase transitions. So far interlayer sliding and anisotropic lattice shrinkage along the stacking orientation of vdW crystals are regarded as the main factors for electronic phase transitions^7–15^. However, for multilayer vdW compounds which consist of a quintuple layer or above, other factors such as atomic motions inside quintuple layer might play a vital role, invoking further thorough investigations.

Here we report the discovery of giant piezoresistivity in *β*′-In_2_Se_3_ owing to the dramatic motion of middle Se atoms inside its quintuple layer. In_2_Se_3_ has long been a well-known multi-functional material with complicated phase space which includes many polymorphs such as the *α, β, γ* and *β*′ phases^16,17^. Recently, several intriguing properties including ferroelectricity, antiferroelectricity, ferroelasticity and paraelectricity have been reported in In_2_Se_3_ by either experiments or theoretical predictions^18–25^. As a result, interests in understanding and searching for these interesting properties in In_2_Se_3_ have been growing rapidly. Hydrostatic pressurization provides an effective way to explore the broad phase space. For example, pressurized *α*-In_2_Se_3_ undergoes the phase transitions in the sequence of *α* → *β*′→*β*, since the increasing pressure can induce anisotropic lattice compression as well as interlayer sliding inside the material^13^. Here we obtain a six-order-of-magnitude drop in the resistivity of *β*′*-*In_2_Se_3_ by applying a small pressure up to 1.2 GPa. The giant change of resistivity results in a large piezoresistive gauge *π*_*p*_ of −5.33 GPa^−1^, which is the largest in vdW crystals so far as we know. In contrast, joint measurements including X-ray diffraction (XRD), Raman spectroscopy and optical linear dichroism microscopy illustrate that the giant resistivity drop is dominated by the shift of middle Se atom in the quintuple layer to the high-symmetric location rather than interlayer sliding or anisotropic lattice shrinkage. First principles density functional theory (DFT) calculations uncover a strong coupling between the band gap size and the ferroelectric dipoles produced by the motions of middle Se atoms.

## Results

### Observation of giant piezoresistivity in *β*′-In_2_Se_3_

**Fig. 1:**
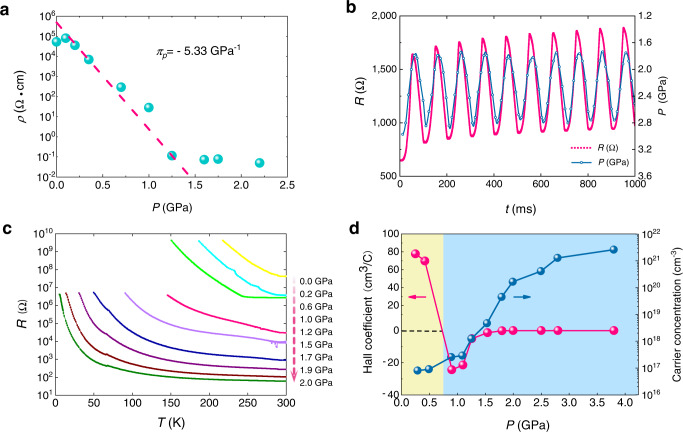
Pressure-induced resistance drop and Hall coefficient measurement of *β*′-In_2_Se_3_. **a** Pressure-dependent resistivity at room temperature. The piezoresistive gauge *π*_*p*_ is estimated from the exponential fit to the data (dashed line) which is a straight line on the semilog plot. **b** Dynamic piezoresistance measurement. **c** Temperature-dependent resistance for pressures of 0 ~ 2.0 GPa. **d** Hall coefficient and carrier concentration as a function of pressure measured at room temperature.

Figure 1a shows the pressure-dependent resistivity of *β*′-In_2_Se_3_ at room temperature. The measurements were carried out using a piston-cylinder cell, see Methods for details. As shown, the effect of hydrostatic pressure on *β*′*-*In_2_Se_3_ is remarkable. Increasing from ambient pressure to about 1.2 GPa, the resistivity drops exponentially over six orders of magnitude. However, above 1.2 GPa, the resistivity only drops slightly. The appearance of the turning point indicates there might be an electronic phase transition at 1.2 GPa. After the full release of pressure, the resistance recovers to the initial 10^5^ Ω·cm according to the direct resistance reading, indicating that the pressure effect is reversible. According to common definition of the piezoresistive gauge (*π*_*p*_), *π*_*p*_ = dlog*ρ*/d*P* (where *P* is the pressure and *ρ* is the resistivity)^26^, the *π*_*p*_ of *β*′*-*In_2_Se_3_ can reach −5.33 GPa^−1^, see Fig. 1a. This value is larger than those of many prominent piezoresistive materials such as SmSe (−5.2 GPa^−1^)^27^, TmTe (−3.7 GPa^−1^)^28^, and Si (−0.2 GPa^−1^)^26^. Such large piezoresistivity actually hasn’t been observed in vdW layered crystals before. For example, many bulk or multilayer vdW crystals such as MoS_2_, WSe_2_, MoSe_2_, and black phosphorous only shows very small piezoresistive gauge^7,9,29,30^. This value is even 20 times larger than that of *α*-In_2_Se_3_^31,32^. The dynamic electrical response to alternating pressure is shown in Fig.1b (see Methods, Supplementary Fig. 1 and Supplementary Note 1 for details). As can be seen, the curve of resistance response follows the curve of pressure variation quite well, which indicates the piezoresistance of *β*′*-*In_2_Se_3_ has a fast response to external pressure. The above results demonstrate that *β*′*-*In_2_Se_3_ is a prominent piezoresistive material which is promising for many electronic applications including strain gauge and wearable device.

Thereafter, we ran temperature-dependent resistance measurements and four-probe Hall measurements to understand the pressure-dependent electronic properties of *β*′*-*In_2_Se_3_, see Methods, Supplementary Fig. 2 and Supplementary Note 2 for details. As shown in Fig. 1c, the material is semiconducting in the pressure range (0~2.0 GPa) as the resistance curves have an increasing trend as the temperature decreases. In another measurement with higher pressure using a diamond anvil cell (DAC) as shown in Supplementary Fig. 3, a decreasing resistance with reducing temperature at high temperature region is observed for pressures above 2.4 GPa, indicating a metallic behavior. Nevertheless, a low-temperature upturn in resistance persists to high pressure, which suggests the survival of the thermal activation across the energy gap. Giving that *β*′*-*In_2_Se_3_ is the indirect band gap semiconductor, the behavior may characterize a transition from semiconductor to semimetal upon compression. Interestingly, the large piezoresistance coincides with this electronic transition. Figure 1d shows the pressure dependence of the Hall coefficient and the carrier density, and shows that the carrier density in *β*′*-*In_2_Se_3_ increases with pressure. In addition, a sign switching of the Hall coefficient (*R*_*H*_) is observed between 0.5 GPa and 0.9 GPa owing to the change of charge carrier from *p* type to *n* type. A hole concentration of 8.98 × 10^16^ cm^−3^ and an electron concentration of 2.56 × 10^17^ cm^−3^ can be estimated from the values of *R*_*H*_ at 0.5 GPa and 0.9 GPa, respectively. A high electron concentration of 2.64 × 10^21^ cm^−3^ is reached at 3.8 GPa. This is consistent with the semiconductor-to-semimetal transition. The Hall carrier density in a semimetal is a mobility-weighted combination of the electron and hole density. The electron mobility is expected to be much higher than hole mobility in *β*′*-*In_2_Se_3_ due to a much smaller effective mass of electrons^33^. So we would expect electron-like Hall effect with high carrier concentration. Similar relation between pressure and *R*_*H*_ has been observed in a vdW semimetal 1*T*-WTe_2_^34^. The change of the sign of *R*_*H*_ in 1*T*-WTe_2_ is observed at a much higher pressure (~10 GPa) due to a significant Fermi surface reconstruction caused by large anisotropic shrinkage of the lattice. Thus, it is important to check the evolution of structure in *β*′*-*In_2_Se_3_ during the pressurization.

### Structure characterizations of *β*′-In_2_Se_3_

**Fig. 2:**
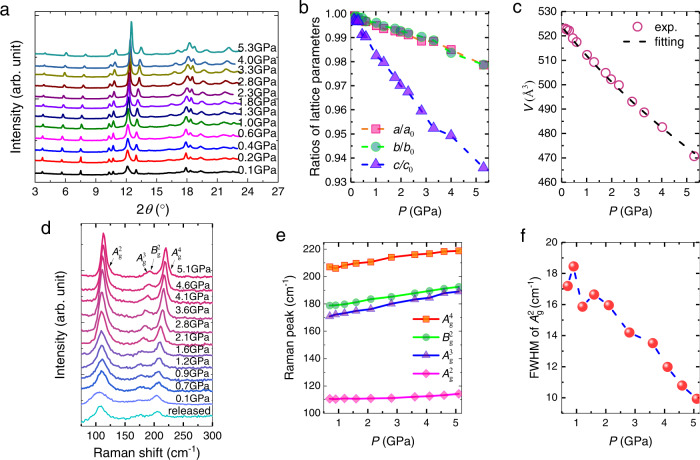
X-ray diffraction (XRD) and Raman measurements of *β*′-In_2_Se_3_. **a** Synchrotron XRD spectra at different pressures. **b** Normalized compression ratio of *a*, *b*, and *c* axis. **c** Volume evolution with pressure extracted from XRD spectra. The black dashed line is a fit to the third-order Birch-Murnaghan equation of state, as described in the main text. **d** Raman spectra at different pressures. **e** Wave vectors of the vibration modes (peaks of the Raman spectra in panel **d)** versus pressure, showing a monotonic increase with pressure with no evidence of a phase transition. The lines are guides to the eye. **f** The FWHM of the ${A}_{g}^{2}$ mode under pressure.

The pressure-lattice relation was obtained by XRD measurements in a DAC (see Methods). Figure 2a presents the pressure-dependent XRD spectra up to 5.3 GPa. As shown, all the XRD spectra have a similar profile except the shifts of diffraction peaks towards higher angles due to the shrinkage of lattice when the pressure increases. Based on the monoclinic cell structure determined by single-crystal XRD (Supplementary Fig. 4), all peaks can exclusively be reproduced by analyzing the data with Le Bail method (Supplementary Fig. 5). Figure 2b shows the anisotropic shrinkage of the lattice. The compression ratio of *c* axis is 1.8% at 1 GPa, almost threefold larger than the *a*-axis compressibility (0.5%). The ratio is much smaller than that in 1*T*-WTe_2_ in which a tenfold anisotropy in shrinkage drives the reconstruction of the Fermi surface^34^. The pressure dependence of volume is shown in Fig. 2c. It is found that the volume decreases smoothly as pressure increases. By fitting the data to the third-order Birch-Murnaghan equation of state (BM-EOS, Supplementary Note 3), we obtained the modulus *K*_0_ = 41.7(1.3) GPa, which is consistent with the results reported previously^13^. Previous study revealed that the increase of pressure can trigger the phase transition from *β*′ to *β* which is associated with a structural change from the monoclinic to the rhombohedral due to interlayer sliding^13^. In present study, the structural transition induced by interlayer sliding is not observed, even though the pressure is up to 5.3 GPa. Thus, the sample should be in *β*′ phase within the pressure range due to the monoclinic structure. After the release of pressure, it can fully recover, see Supplementary Fig. 6.

To further confirm the structural phase in the pressure range, Raman scattering measurements were carried out, since several phonon vibrational modes of In_2_Se_3_ are dependent on its phase^13,14,35,36^. The pressure-dependent Raman spectra are plotted in Fig. 2d. Characteristic vibration modes of *β*′ phase are observed clearly within the studied pressure range up to 5.1 GPa. Continuous blue shifts of the Raman peaks are observed when the pressure increases (Fig. 2e). The sample can recover after the release of pressure. Meanwhile, Fig. 2f indicates that the full width at half-maximum (FWHM) of ${A}_{g}^{2}$ phonon decreases continuously and suggests no phase transition^13^. Moreover, when we extended the experiment to higher pressures, the vibration peak ${A}_{g}^{4}$ mode shifts to lower frequency abruptly at 11.2 GPa which should be attributed to the emergence of the *E*_g_ mode of the *β* phase, see Supplementary Fig. 7. The critical pressure is close to the one reported by Vilaplana et al.^13^. Thus, by using Raman spectroscopy, we confirm that the sample remains in the *β*′ phase and the large piezoresistivity cannot be ascribed to structural phase transition.

### Pressure induced evolution of linear dichroism

**Fig. 3:**
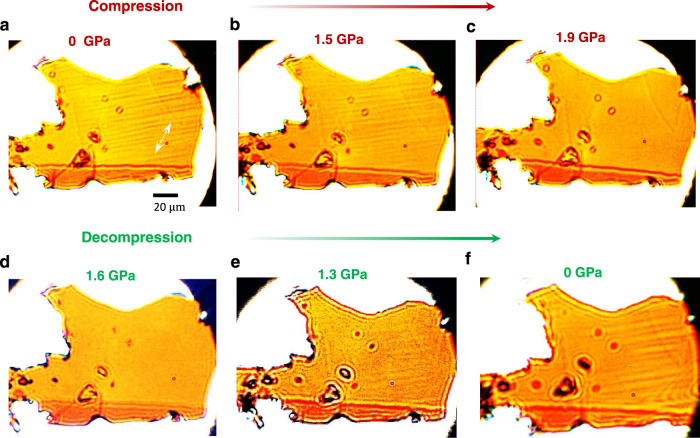
Pressure-dependent linear dichroism of *β*′-In_2_Se_3_. **a–c** Micrographs of linear dichroism take as pressure increases from 0 GPa to 1.9 GPa, showing disappearance of the observable domain contrast (narrow strips in **a** and **b**). The double-arrow symbol (white color) indicates the direction of linear polarization. **d–f** Recovery of the domain contrast upon releasing the pressure.

The *β*′ phase In_2_Se_3_ is characterized by a well-known striped superstructure formed by the atomic deviation of middle Se atoms from the central location in the quintuple layer^21,22,24,25^. The striped superstructure exhibits a strong linear dichroism and results in remarkable domain contrast in polarized light imaging^25^. We thus investigate the pressure-dependent linear dichroism of *β*′*-*In_2_Se_3_ using a linear polarized light microscope together with a DAC, see Methods. Figure 3a–c presents the optical images of a thin *β*′*-*In_2_Se_3_ flake under compression. To ensure good light transmission, the sample thickness should be much less than 1 μm, and strong image contrast of different domains can be observed. The domains appear as narrow strips about 100 μm in length and <5 μm in width. The domain contrast becomes weaker as the pressure increases to 1.5 GPa and even invisible at 1.9 GPa. The absence of domain contrast at 1.9 GPa is confirmed by rotating the polarizer (Supplementary Fig. 8). The result indicates the loss of linear dichroism under pressure. The domain contrast recovers when the sample is decompressed to zero pressure as shown in Fig. 3d–f. The observations suggest that the compression plays a significant role in shifting the middle Se atom towards the high symmetry point and weakening the striped superstructure. The phase transition from semiconductor to semimetal is observed near the critical pressure that linear dichroism disappears, indicating that the transition is associated with the shifting of middle Se atom.

### Modeling the pressure induced evolution of band structure

**Fig. 4:**
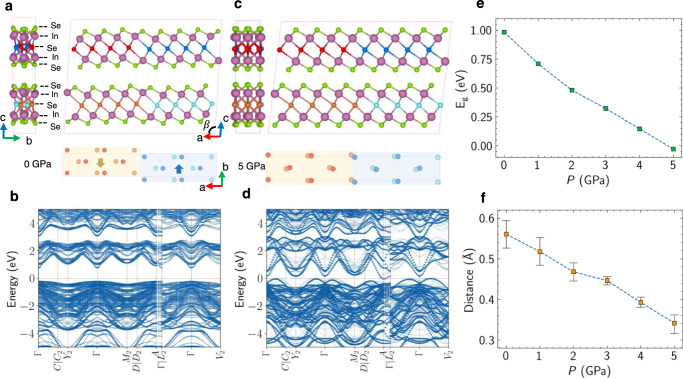
Density functional theory calculations for pressure induced changes in band structure. **a, c** Calculated atomic structures, and **b, d** band structures of *β*′*-*In_2_Se_3_ at 0 GPa and 5 GPa, respectively. The bottom panels in **a** and **c** only plot the middle Se atoms. The large solid arrows in **a** indicate ferroelectric dipoles which disappear at 5 GPa since it becomes semimetal. **e** Evolution of band gap (*E*_*g*_) as a function of pressure. **f** The distance between middle Se atoms and high-symmetry locations as a function pressure. The scale bar defines the maximum and the minimum distances of the middle Se atoms to high-symmetry positions at each pressure.

To gain further insights into the effect of pressure on the band structure of *β*′*-*In_2_Se_3_, we performed first principles DFT calculations using a 1 × 8 superstructure constructed by shifting two groups of middle Se atoms reversibly along the *b* axis and then relaxing the superstructure to a stable state, see Fig. 4a. The atomic model is constructed based on Xu’s work and the shifts of middle Se atoms away from high-symmetry locations produce antiparallel ferroelectric dipoles^37^. For comparison, the middle Se atoms without shifting away from high-symmetry locations are shown in Supplementary Fig. 9. Figure 4b shows the band structure of the *β*′ superstructure at 0 GPa which indicates a sizable indirect band gap as well as the *p*-type doping. As the pressure increases, the middle Se atoms move towards the high-symmetry positions and the band gap decreases dramatically due to the substantial downward movement of the conduction band at *A* point, see Fig. 4c, d. The conduction band minimum (CBM) moves across the Fermi level so quick that the dominating carriers for transport switch from hole-like to electron like. The result is consistent with the change of the sign of the Hall coefficient in Fig. 1d. The calculated band gap size as a function of pressure is shown in Fig. 4e. The band gap size is calculated as the energy difference between CBM and valance band maximum (VBM). The negative band gap size means the overlap between CBM and VBM, see Fig. 4d for example. The indirect band gap closure in Fig. 4d is also in good agreement with the pressure-induced semiconductor-to-semimetal transition shown in Fig. 1. It should be pointed out that DFT calculation is carried out at zero temperature. Thus, much larger pressures are required to close the band gap in calculation when compared with experiments done at finite temperatures^38^. This would enlarge the influence of lattice shrinkage on the band structure modification in DFT calculations comparing to experiments. Figure 4f plots the pressure-induced motions of middle Se atoms of the superstructure. In the plot, the distance is defined as the length between the middle Se atom and its corresponding high-symmetry position. The coordinates of the high-symmetry position of each middle Se atom are calculated by averaging the coordinates of its six nearest In atoms. Similarly, pressure induced band gap closure is observed in our DFT calculations based on the unit cell model, see Supplementary Fig. 10. It should be noticed that the middle Se atoms shift away from high-symmetry locations along *a* axis after lattice relaxation. The different shifting directions of middle Se atoms between the superstructure and the unit cell indicate that the band gap size is controlled by the distance between middle Se atom and its high-symmetry position rather than the shifting direction. Supplementary Fig. 11 indicates that the band gap changes from 0.81 eV to 0.29 eV after shifting middle Se atoms only. In contrast, the lattice shrinkage only changes the band gap from 0.81 eV to 0.54 eV as the pressure increases from 0 GPa to 5 GPa (Supplementary Fig.12). All the results indicate that intralayer motions of middle Se atoms contribute significantly to the band gap closure, while lattice shrinkage has minus influence.

Since shifting middle Se atoms away from high-symmetry positions produces ferroelectric dipoles (antiferroelectricity) and symmetry-equivalent strain (ferroelasticity)^22,37^, the middle Se atoms moving towards high-symmetry positions upon compression can help release the spontaneous strain and thereafter weaken the ferroelectric dipoles. Different to *α*-In_2_Se_3_ and perovskite ferroelectrics^31,32,39^, the existence of giant piezoresistivity in *β*′*-*In_2_Se_3_ implies a strong coupling between band structure, antiferroelectricity and ferroelasticity.

## Discussion

The compression ratio along the *a, b* and *c* orientations are less than 2% over the low-pressure range where a rapid drop of resistance is found, suggesting that reduction of geometric dimension is not responsible for the large piezoresistivity. The metallic behavior above 2.4 GPa is the signature of band gap closure, which is confirmed qualitatively by the DFT calculation in Fig. 4. With the band gap closing, the carrier concentration increases by almost five orders of magnitude. Therefore, the band gap shrinkage dominates the large piezoresistivity. In a compressed vdW material, the electronic structure is commonly modified by the anisotropic lattice shrinkage along the stacking orientation or the interlayer sliding. However, interlayer sliding can be ignored at least at pressure below 5.3 GPa for *β*′*-*In_2_Se_3_ because the$\,\beta^ {\prime}$-to-*β* phase transition corresponding to the interlayer sliding, takes place at much higher pressure (~11.2 GPa)^13^. In contrast, the large piezoresistive effect occurs at the pressure just slightly before the stripe superstructure of middle-Se-atom displacements is suppressed, indicating a strong correlation between the piezoresistance and motion of middle Se atoms to the central position to release symmetry-equivalent strain and thereafter weaken the in-plane ferroelectric dipoles. All these results conclude unambiguously that the motion of the middle Se atoms plays a dominating role in the large piezoresistive effect.

In summary, we have reported a large piezoresistivity in a vdW layered crystal *β*′-In_2_Se_3_. The strong link between the piezoresistivity and the pressure induced motion of the middle Se atoms is confirmed by pressure-dependent XRD, Raman spectra, linear dichroism studies as well as first principles DFT calculations. This study emphasizes the contrast motions of different atomic layers within a quintuple layer, which is very different from previous studies on vdW crystals of which lattice shrinkage and interlayer sliding play a central role on their changes of electronic properties under pressure^7,10,34^. Our results thus not only discover a vdW layered material possessing excellent piezoresistivity, but also reveal an extra degree of freedom in vdW materials to modulate the electronic properties by pressure such as band structure and ferroelectric dipoles.

## Methods

### Sample preparation

Well characterized high quality single crystals of *β*′-In_2_Se_3_ were purchased from HQ Graphene. Fresh thin flakes exfoliated from the crystals were used for electrical transport, Raman and linear dichroism measurements. Clean crystal was ground to powder for high pressure synchrotron XRD measurement.

### Structural and Raman measurements

The in-situ XRD experiments were performed at beamline 4W2 at Beijing Synchrotron Radiation Facility. The wavelength was 0.6199 Å. The single-crystal XRD was carried out on a Bruker D8 Venture diffractometer with PHOTON III detector in shutterless mode with an Incoatec Microfocus source (Mo-Diamond Kα radiation, λ = 0.71073 Å). The monoclinic cell structure parameters were deduced from the XRD pattern using APEX4 software. Pressure-dependent Raman spectra were collected on a commercial Raman spectrometer of HORIBA LabRAM HR evolution by using a 532 nm laser with continuous wave mode. Symmetric DAC were used for the XRD and Raman measurements. Silicone oil was used as the pressure-transmitting medium (PTM) for the XRD experiments and liquid Argon was used for the Raman measurements. The pressure was calibrated according to the fluorescence of the ruby powder. The unit cell parameter refinements were performed with GSAS software.

### Electrical transport measurements

Piston cylinder cell (PCC) was used to measure the pressure-dependent sheet resistance of the *β*′-In_2_Se_3_ flakes in low pressure region (<2.5 GPa). Daphne 7373 was used as PTM, and the pressure was determined by the superconducting Pb. A nonmagnetic copper beryllium DAC was used to probe the sheet resistance and Hall measurement in order to extend to the study to higher pressure (>2.5 GPa). Silicone oil was used as PTM, and the pressure was calibrated by the ruby powder. The resistivity and Hall voltage were measured in four-probe geometry in a Physical Property Measurement System (PPMS, Quantum Design).

### Dynamical resistance measurements

The resistance under the dynamic effort of pressure was measured by the combination of the dynamic loading device (DLD) and the traditional high-pressure resistance measuring method. The detailed introduction of DLD can be found in the Supplementary Fig. 1. The assembling of sample is similar to the traditional resistance measurements. Four microelectrodes were fabricated on the anvil in the piston by micro-fabrication technology. The voltage signals during the dynamic (de)compression were collected using PCL-5922 digitizer. The pressure was calibrated according to the fluorescence of the ruby. NaCl was loaded as the PTM.

### Linear dichroism measurements

The exfoliated flakes were loaded in a symmetric DAC with 500 μm diameter culet-sized diamond anvils. Silicone oil was used as the PTM. Linear polarized light was incident from the bottom of the DAC, passed through the sample, and the domain images were captured by a CCD at the top of the microscopy.

### First-principles DFT calculations

First-principles Density Functional Theory (DFT) calculations were conducted using Vienna Ab-Initio Simulation Package (VASP). All structures were fully relaxed until the forces between ions are less than 0.01 eV/Å. The ionic relaxations and band structure calculations were performed at Perdew− Burke−Ernzerhof (PBE) level with the exchange-correlation potential approximated by the generalized gradient approximation (GGA). The Brillouin Zone of superstructure and unit cell In_2_Se_3_ is sampled with 1 × 6 × 1 and 21 × 21 × 3 kpoints grid, respectively. The energy cut off was set to 400 eV.

### Reporting summary

Further information on research design is available in the Nature Portfolio Reporting Summary linked to this article.

## Supplementary information

Supplementary information

Lasing Reporting Summary

Reporting Summary

## Data Availability

All relevant data in this study are included in the article and its Supplementary Information, and are available from the authors upon request. The source data is provided with this work as a Source Data file. Source data are provided with this paper.

## Source data

Source Data
