# Supplementary material for: Giant piezoresistivity in a van der Waals material induced by intralayer atomic motions
Source: Nat Commun. 2023 Mar 18;14:1519. doi: 10.1038/s41467-023-37239-9 (PMC10024745; doi:10.1038/s41467-023-37239-9)
Supplement: Supplementary file 1 — Supplementary information [file 41467_2023_37239_MOESM1_ESM.pdf]

Supplementary Information for

Giant piezoresistivity in a van der Waals material induced by intralayer atomic motions

Lingyun Tang[1,#], Zhongquan Mao[1,#], Chutian Wang[2,#], Qi Fu[3], Chen Wang[3], Yichi Zhang[3], Jingyi Shen[3], Yuefeng Yin[2], Bin Shen[4], Dayong Tan[5], Qian Li[6], YonggangWang[7], Nikhil V. Medhekar[2], Jie Wu[3], Huiqiu Yuan[4], Yanchun Li[8*], Michael S. Fuhrer[9*], Changxi Zheng[3*]

[1]School of Physics and Optoelectronics, South China University of Technology, Guangzhou, Guangdong 510641, China

[2]Department of Materials Science and Engineering, & ARC Centre of Excellence in Future Low Energy Electronics Technologies, Monash University, Clayton, VIC 3800, Australia.

[3]Key Laboratory for Quantum Materials of Zhejiang Province, School of Science, Westlake University, Hangzhou, Zhejiang 310024, China

[4]Center for Correlated Matter and Department of Physics, Zhejiang University, Hangzhou, Zhejiang 310024, China

[5]Guangzhou Institute of Geochemistry, Chinese Academy of Sciences, 510640, Guangzhou, Guangdong, China

[6]Shandong Key Laboratory of Optical Communication Science and Technology, School of Physics Science and Information Technology, Liaocheng University, Liaocheng 252000, China

[7]Center for High Pressure Science and Technology Advanced Research (HPSTAR), Beijing 100094, China

[8]Institute of High Energy Physics, Chinese Academy of Sciences, Beijing 100049, China

[9]ARC Centre of Excellence in Future Low-Energy Electronics Technologies, & School of Physics and Astronomy, Monash University, Victoria 3800, Australia

[#]These authors contributed equally: Lingyun Tang, Zhongquan Mao, Chutian Wang.

[*]Email: zhengchangxi@westlake.edu.cn; michael.fuhrer@monash.edu;

liyc@ihep.ac.cn

Supplementary Figures

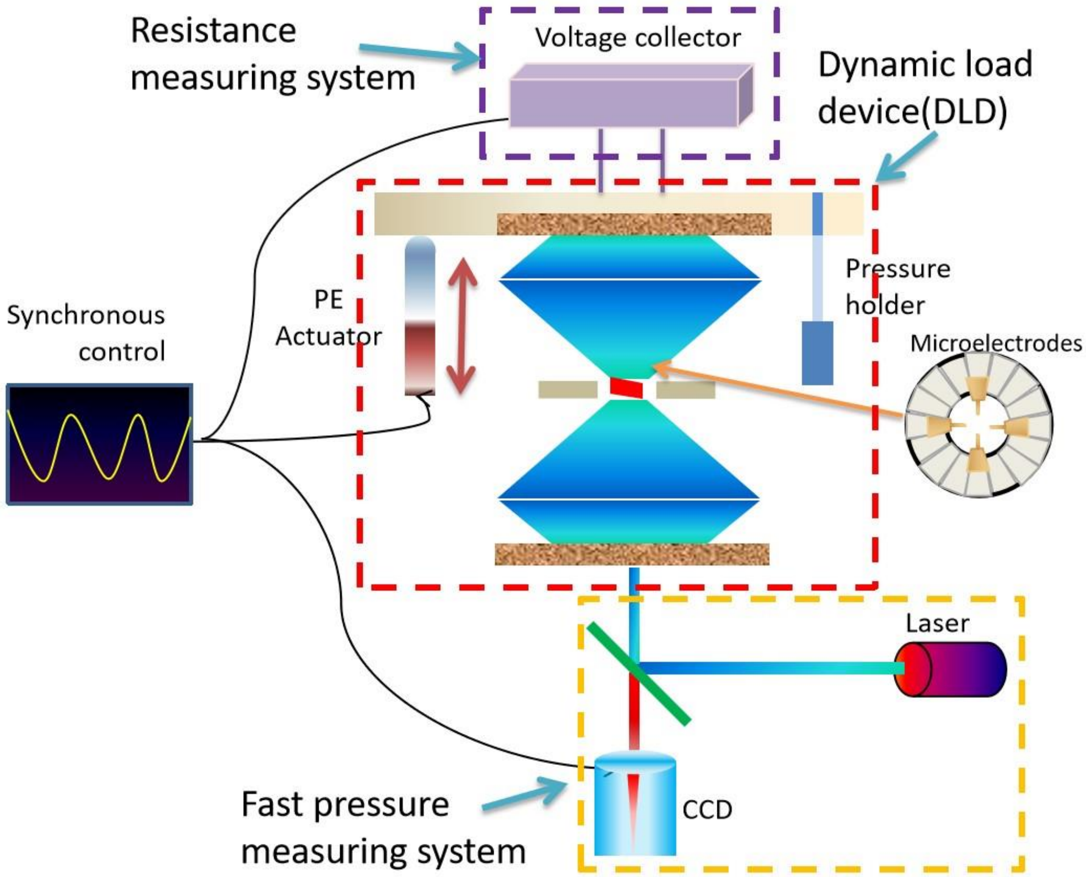

Supplementary Figure 1 | Dynamic electrical measurement system. A schematic diagram of the system for measuring the dynamic resistance response of β'-In_2Se_3 to pressure. The system includes three parts: (1) the dynamic loading device (DLD) assembling with a symmetric diamond anvil cell (DAC)[1] (red dashed box). Four microelectrodes were fabricated on the anvil in the piston by micro-fabrication technology[2]. (2) fast pressure measuring system (orange dashed box), and (3) resistance measuring system (purple dashed box). The three parts are synchronously controlled by an arbitrary-wave function generator.

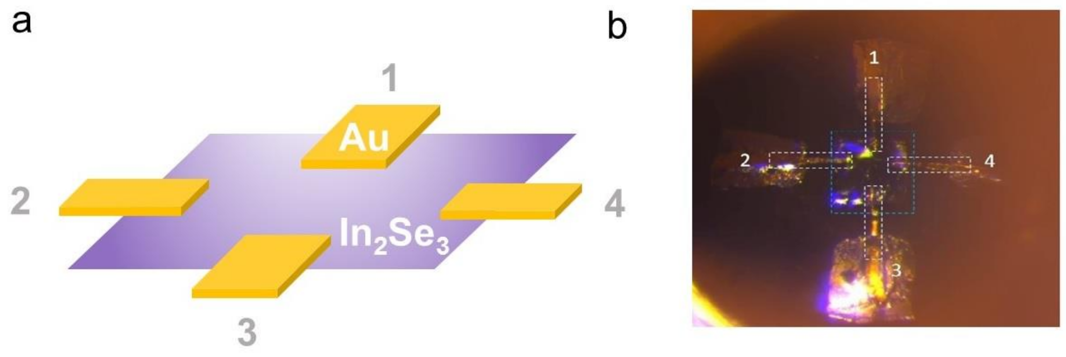

Supplementary Figure 2 | Hall measurements. (a) The electrode configuration of four probes used for Hall coefficient (R_H) measurements. (b) The image of a typical sample measured in a DAC. The white rectangles numbered with 1,2,3,4 indicate the gold electrodes, and the blue square denotes the β'-In_2Se_3 sample.

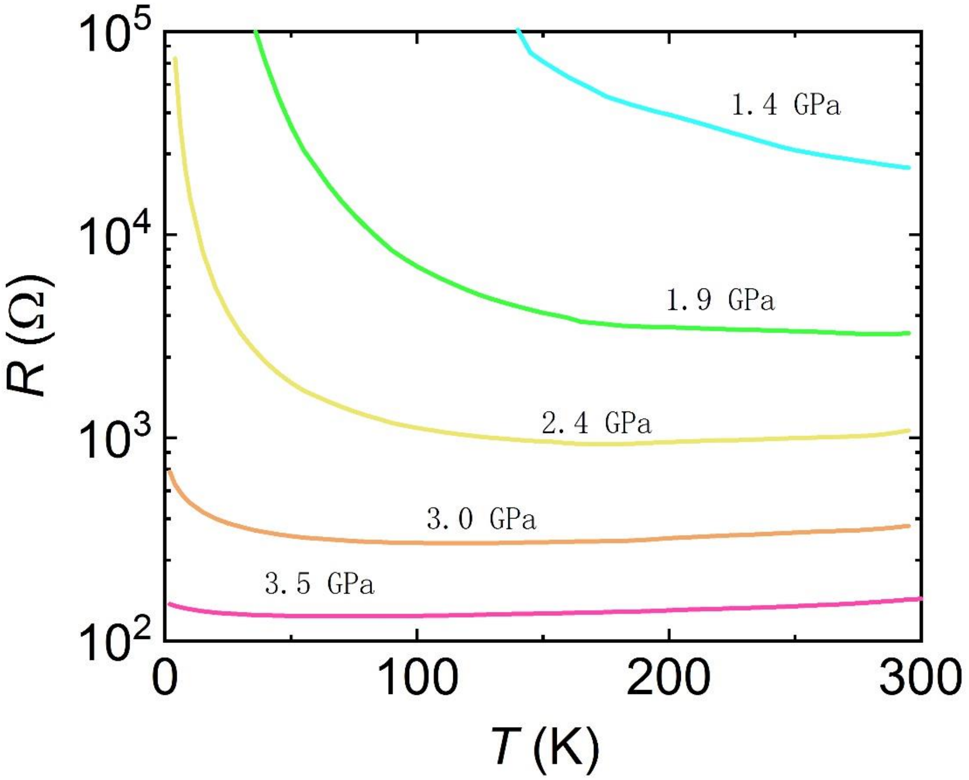

Supplementary Figure 3 | Temperature dependent resistance for extended pressure. The curves were obtained in a DAC in order to extend measurement to higher pressure.

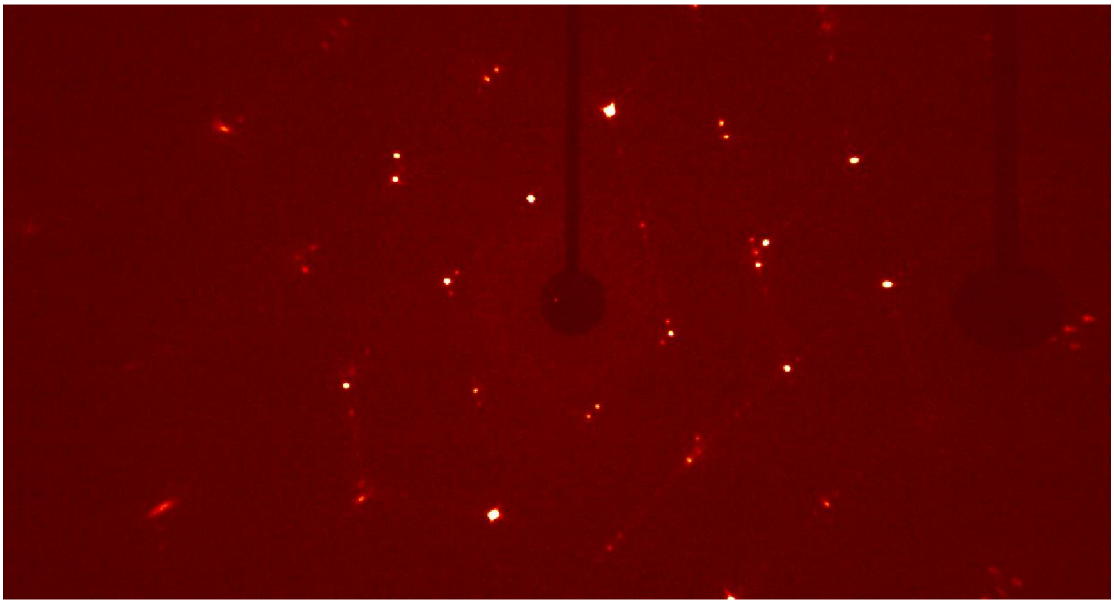

Supplementary Figure 4 | Single-crystal XRD pattern of β'-In_2Se_3. The diffraction pattern is taken at ambient conditions. Deduced from the pattern, the crystal has a monoclinic cell structure with lattice parameters: *a*= 6.93Å, *b*= 3.98Å, *c*= 19.43Å, α= 90.00°, β= 103.25°, γ= 90.00°. As shown, the superstructural diffraction spots are not strong enough to deduce the superstructure of β'-In_2Se_3. The sample was made from the same big crystal for other experiments.

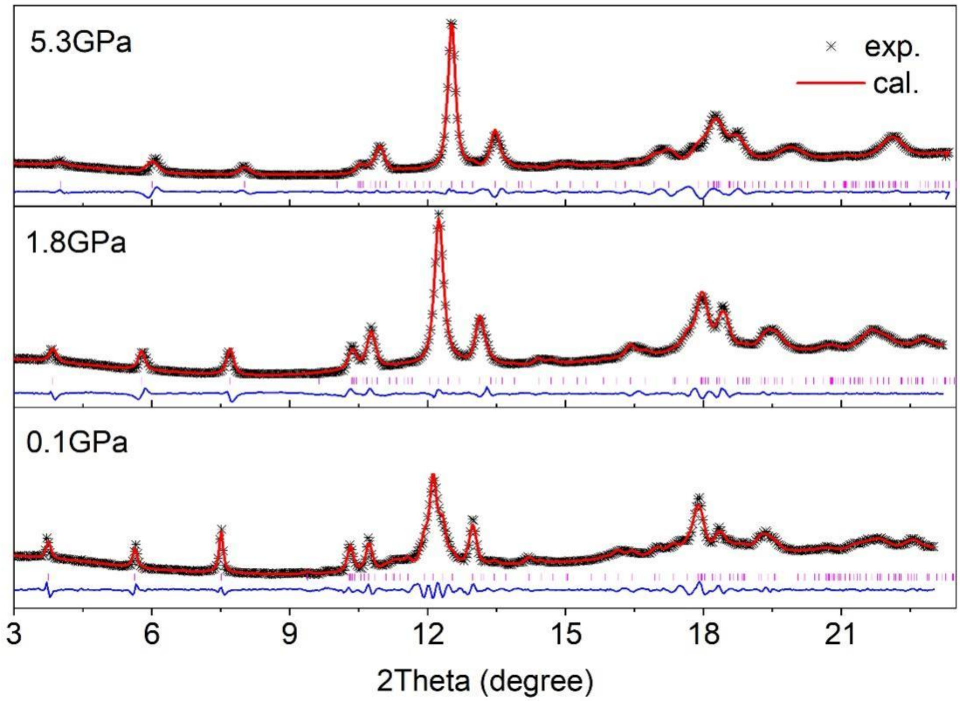

Supplementary Figure 5 | Le Bail fitting of the XRD spectra. The Lebail profile fitting patterns in β'-In_2Se_3 with monoclinic structure under 0.1 GPa, 1.8 GPa, and 5.3 GPa. The black aterisks are the experimental data points, the red lines represent the fitting patterns, the magenta stick marks are the calculated Bragg reflections and the residuals are shown by the blue lines.

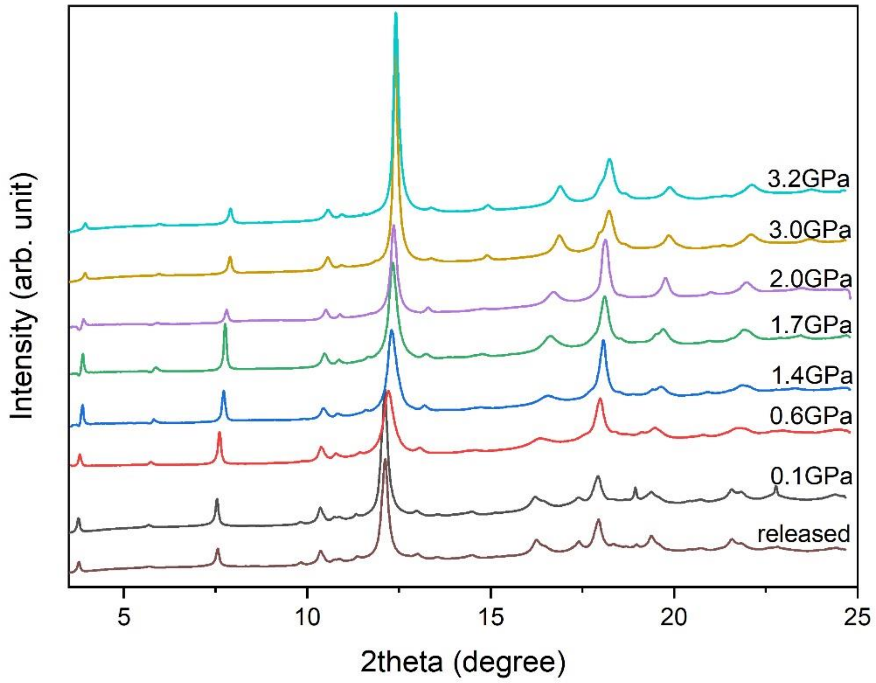

Supplementary Figure 6 | XRD spectra of β'-In_2Se_3 powder. The XRD spectra of the sample are taken in a pressurization-released cycle. The sample can recover after pressure is released. The experiment was carried out on the beamline BL15U at Shanghai Synchrotron Radiation Facility. The sample was made from the same big crystal for other experiments.

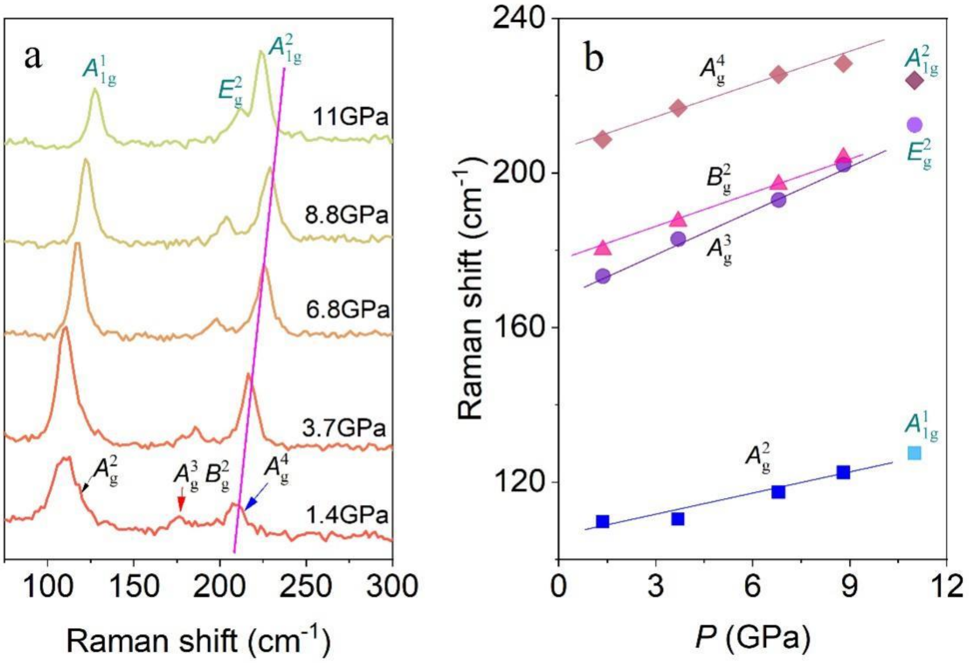

Supplementary Figure 7 | Raman spectra at high pressures. **a** The Raman spectra of β'-In_2Se_3 at different pressures, and **b** the pressure dependence of experimental Raman-active mode frequencies of four vibration modes A_g^2, A_g^3, B_g^2, A_g^4. The solid lines are guidance for eyes.

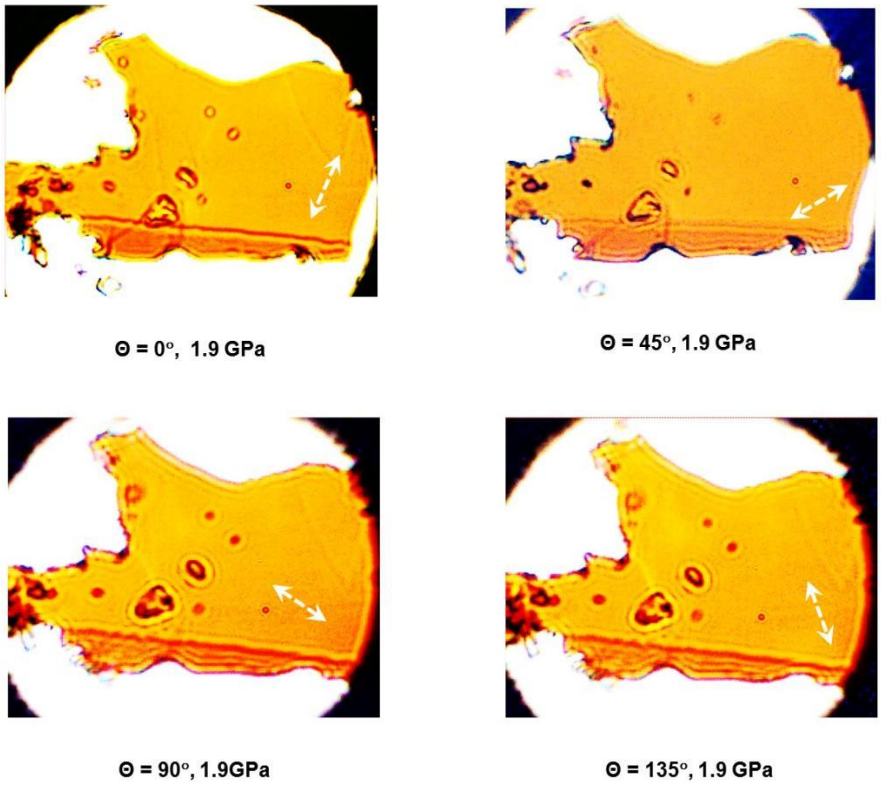

Supplementary Figure 8 | Linear polarized optical imaging. The sequential optical microscopy images of β'-In_2Se_3 pressurized at 1.9 GPa are obtained by using the linear polarized light along different directions. The white double arrow denotes the polarization direction. No domain contrast is observed in the images.

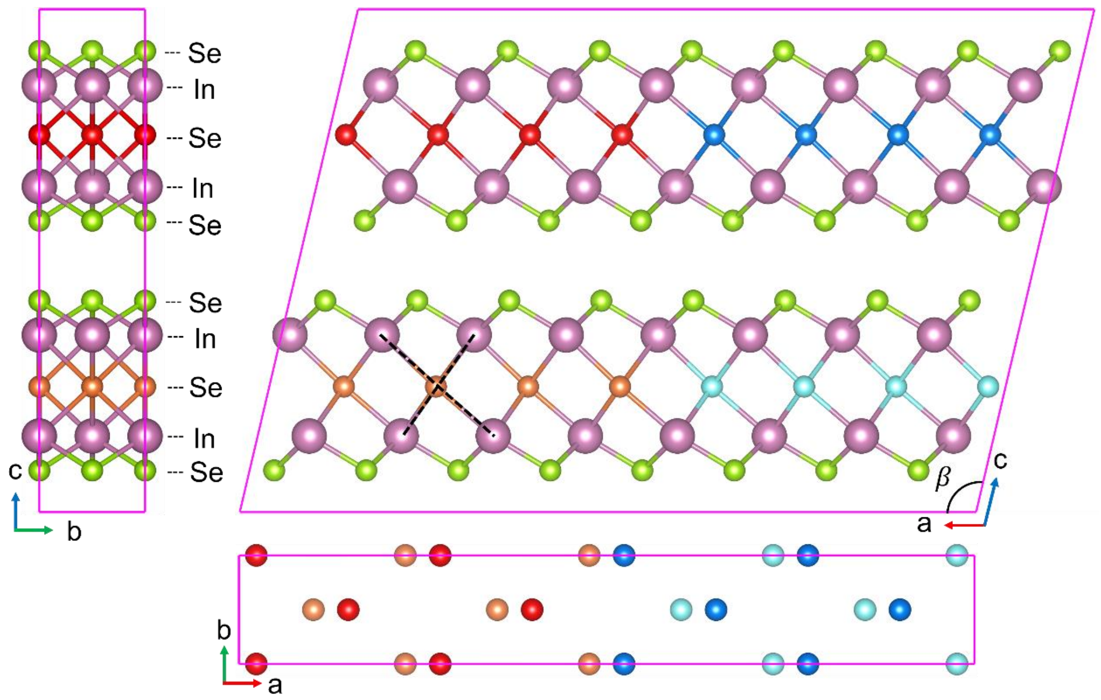

Supplementary Figure 9 | In_2Se_3 superstructure with middle Se atoms at high-symmetry locations. The 1X8 superstructure is directly extended from the monoclinic unit cell structure determined by the single-crystal XRD measurement. As shown by the dashed black lines, the middle Se atoms are at high-symmetry locations. The bottom panel indicates middle Se atoms only.

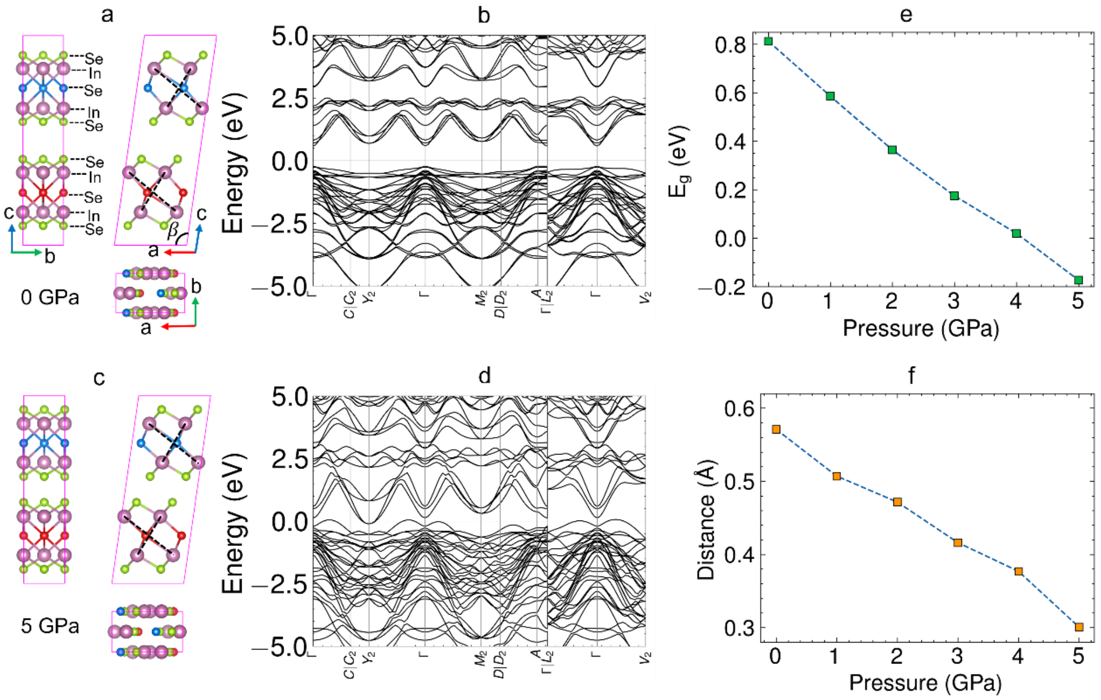

Supplementary Figure 10 | DFT calculations for β'-In_2Se_3 with unit cell structure. The shifts of middle Se atoms are obtained by directly relaxing the monoclinic cell structure obtained by the single-crystal XRD measurement. **a** The atomic model of the β'-In_2Se_3 at 0 GPa. As shown, the middle Se atoms shift away from the high-symmetry points along *a* axis. **b** The corresponding band structure at 0 GPa. **c** the atomic structure at 5 GPa, indicating the middle Se atoms is closer to the high-symmetry points. **d** The corresponding band structure at 5 GPa indicating a band gap closure. **e** The band gap size (E_g) as a function of pressure. **f** The distance between middle Se atoms and the high-symmetry points as a function of pressure.

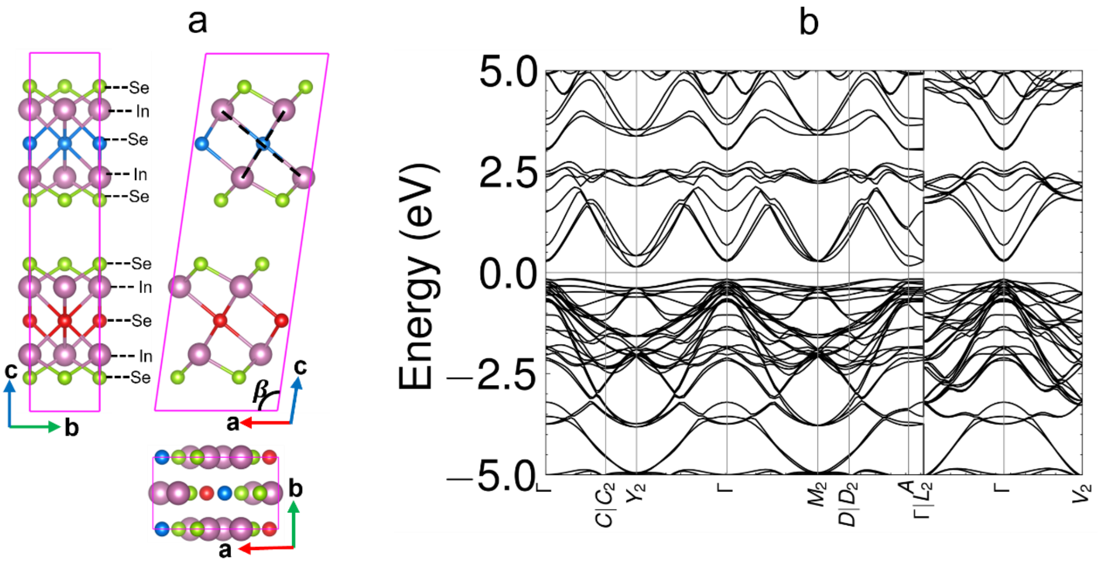

Supplementary Figure 11 | Band structure for β'-In_2Se_3 at 0 GPa with middle Se atoms at high-symmetry locations. a The atomic model is obtained by shifting the middle Se atoms of the atomic model shown in Supplementary Fig. 10a to the high-symmetry locations. **b** The corresponding band structure indicates a 0.29 eV band gap. The result illustrates that the band gap changes from 0.81 eV to 0.29 eV by shifting the middle Se atoms only.

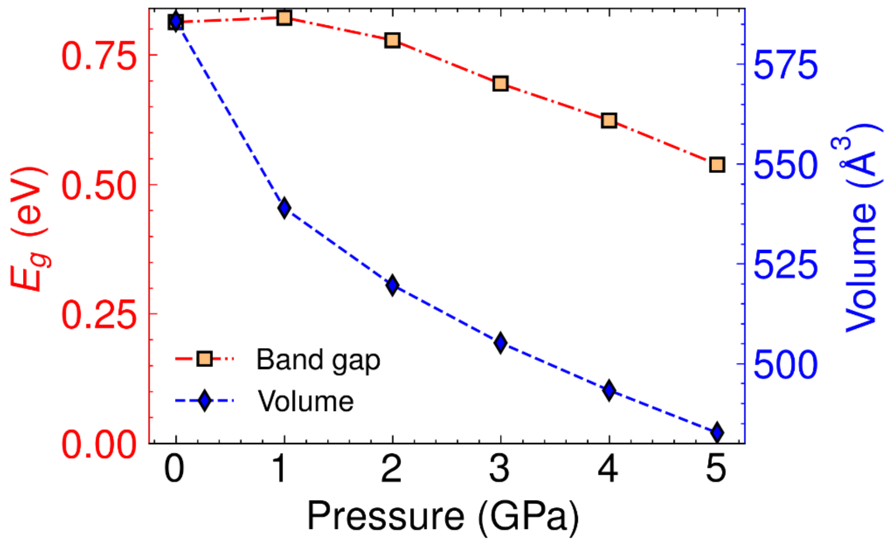

Supplementary Figure 12 | Effect of lattice shrinkage on the band gap (E_g) and the volume. The DFT calculations were carried out by directly shrinking the atomic structure of β'-In_2Se_3 shown in Supplementary Figure 10a to each desired pressure.

Supplementary Notes

Supplementary Note 1: Dynamic resistance measurement. The dynamic response of resistance under pressure was measured in a high-pressure dynamic measurement system, as shown in Supplementary Figure 1. In the experiments, the sample was compressed up to around 3 GPa at first. The data collecting process was starting in decompressing run repeating as decompressing → compressing → decompressing → compressing→ ··· and modulated by the periodic signals in a pressure range of 3 GPa to lower pressure.

Supplementary Note 2: Details of Hall measurement

The Hall coefficient (R_H) are measured using Van der Pauw Geometry as shown in Supplementary Figure 2. In order to exclude the contribution due to longitudinal misalignment voltage and thermoelectric effects, such as Nernst effect, the direction of applied field and current were reversed at each given temperature and field. The Hall voltage was then derived from the antisymmetric part of the transverse voltage.

Supplementary Note 3: Le Bail fitting and Birch-Murnaghan equation of state. The XRD data were analyzed by Le Bail refinement using the GSAS software. All the data at pressure up to 6.9 GPa can be well reproduced by the monoclinic structure with space group *C*2/m, as shown in Supplementary Figure 5. The volume V was fitted to the third-order Birch-Murnaghan equation of state(BM-EOS): $P = \frac{3}{2}K_0\left[\left(\frac{V_0}{V}\right)^{\frac{7}{3}} -\right.$

$\left(\frac{V_0}{V}\right)^{\frac{5}{3}}\Bigg] \times \left\{1 + \frac{3}{4}(K_0' - 4)\left[\left(\frac{V_0}{V}\right)^{\frac{2}{3}} - 1\right]\right\}$, where K_0 and K' is the bulk modulus and first pressure derivative of the bulk modulus at zero pressure, respectively. V_0 is the volume at ambient pressure, and P is the pressure.

References

[1] Cheng H. *et al*. A convenient dynamic loading device for studying kinetics of phase transitions and metastable phases using symmetric diamond anvil cells. *High Pressure Research* 38, 32-40 (2018).

[2] Liu J. *et al*. Fabrication of microelectrodes on diamond anvil for the resistance measurement in high pressure experiment. *Microsystem Technologies* 24, 3193-3199 (2018).
