# Supplementary material for: Giant piezoresistivity in a van der Waals material induced by intralayer atomic motions
Source: Nat Commun. 2023 Mar 18;14:1519. doi: 10.1038/s41467-023-37239-9 (PMC10024745; doi:10.1038/s41467-023-37239-9)
Supplement: Supplementary file 2 — Lasing Reporting Summary [file 41467_2023_37239_MOESM2_ESM.pdf]

nature research

Corresponding author(s): Changxi Zheng

Lasing Reporting Summary

Nature Research wishes to improve the reproducibility of the work that we publish. This form is intended for publication with all accepted papers reporting claims of lasing and provides structure for consistency and transparency in reporting. Some list items might not apply to an individual manuscript, but all fields must be completed for clarity.

For further information on Nature Research policies, including our data availability policy, see Authors & Referees.

ü Experimental design

Please check: are the following details reported in the manuscript?

1. Threshold

Item	Answer	Details
Plots of device output power versus pump power over a wide range of values indicating a clear threshold	☐ Yes ☒ No	This is not involved in this manuscript.

2. Linewidth narrowing

Item	Answer	Details
Plots of spectral power density for the emission at pump powers below, around, and above the lasing threshold, indicating a clear linewidth narrowing at threshold	☐ Yes ☒ No	This is not involved in this manuscript.
Resolution of the spectrometer used to make spectral measurements	☐ Yes ☒ No	This is not involved in this manuscript.

3. Coherent emission

Item	Answer	Details
Measurements of the coherence and/or polarization of the emission	☐ Yes ☒ No	This is not involved in this manuscript.

4. Beam spatial profile

Item	Answer	Details
Image and/or measurement of the spatial shape and profile of the emission, showing a well-defined beam above threshold	☐ Yes ☒ No	This is not involved in this manuscript.

5. Operating conditions

Item	Answer	Details
Description of the laser and pumping conditions *Continuous-wave, pulsed, temperature of operation*	☒ Yes ☐ No	Page 15
Threshold values provided as density values (e.g. W cm^{-2} or J cm^{-2}) taking into account the area of the device	☐ Yes ☒ No	This is not involved in this manuscript.

6. Alternative explanations

Item	Answer	Details
Reasoning as to why alternative explanations have been ruled out as responsible for the emission characteristics *e.g. amplified spontaneous, directional scattering; modification of fluorescence spectrum by the cavity*	☐ Yes ☒ No	This is not involved in this manuscript.

7. Theoretical analysis

Item	Answer	Details
Theoretical analysis that ensures that the experimental values measured are realistic and reasonable *e.g. laser threshold, linewidth, cavity gain-loss, efficiency*	☐ Yes ☒ No	This is not involved in this manuscript.

8. Statistics

Item	Answer	Details
Number of devices fabricated and tested	☐ Yes ☒ No	This is not involved in this manuscript.
Statistical analysis of the device performance and lifetime (time to failure)	☐ Yes ☒ No	This is not involved in this manuscript.
